# Dentists’ Competence and Knowledge on Domestic Violence and How to Improve It—A Review

**DOI:** 10.3390/ijerph19074361

**Published:** 2022-04-05

**Authors:** Jana Lauren Bregulla, Marcel Hanisch, Bettina Pfleiderer

**Affiliations:** 1 Department for Prosthodontics and Biomaterials, Albert-Schweitzer-Campus 1, University Hospital Muenster, Building W 30, 48149 Muenster, Germany; jana.bregulla@ukmuenster.de; 2 Clinic for Radiology, Albert-Schweitzer-Campus 1, University Hospital Muenster, Building A 16, Medical Faculty University of Muenster, 48129 Muenster, Germany; 3 Research Unit Rare Diseases with Orofacial Manifestations, Department of Cranio-Maxillofacial Surgery, Albert-Schweitzer-Campus 1, University Hospital Muenster, Building W 30, 48149 Muenster, Germany; marcel.hanisch@ukmuenster.de

**Keywords:** domestic violence, intimate partner violence, dentistry, domestic violence education, screening

## Abstract

Domestic violence (DV) is an important public health topic with a high prevalence in society. Dentists are also frontline responders to DV, as they not only treat victims of DV with dental injuries, but they can also screen for the presence of DV because they see patients for regular check-ups. Using the WHO definition, which describes domestic violence as intimate partner violence, 17 papers could be included in our analyses. The results of this review clearly indicated that although dentists, as members of the health care sector, are important frontline responders to DV, they are neither trained adequately at medical school nor do most feel competent enough to ask victims about DV or support them as needed. DV is often not taught at dentistry schools at all. The aims of this review were to provide an overview of existing literature on dentists’ knowledge and beliefs regarding DV, whether and how DV is taught in medical education and to give recommendations on how to improve the education of dentists on this topic. Based on our findings, we recommend that DV education should be mandatory at dentistry schools and in further training for dentists with a focus on communication with victims, how DV can be identified and how to support victims well.

## 1. Introduction

Since the outbreak of COVID-19, domestic violence (DV) came increasingly into the limelight [1], as many people were forced to stay mostly at home and live socially distanced due to quarantine measures [1]. Recent data have indicated that DV against woman is on the rise [2], in particular the numbers of calls to shelters and helplines have increased during the COVID-19 pandemic [3]; for example, case numbers rose by 18% in Spain and by 30% in France in early 2020 [4]. It soon became clear that the detection and support of victims as well as the first line response to DV need to be improved. As dentists also see DV victims, we wanted to obtain a better understanding about their knowledge on the topic of DV, their competence in dealing with victims and whether there was any training on DV available to them by reviewing the existing publications in this field.

We used the WHO definition of domestic violence as intimate partner violence and as a “pattern of behaviour in any relationship that is used to gain or maintain power and control over an intimate partner” [5].

What starts with humiliation and manipulation might end in hurting and injuring the abused person. Different forms of DV, such as sexual, physical, emotional and economic violence, can occur [5]. A distinction is made between short-, medium- and long-term consequences: short- time consequences are physical injuries, which in the worst case could lead to death. The medium- and long-term effects of DV on health can result in a range of psychological and psychosomatic symptoms [6]. Moreover, it is important to keep in mind that about 28–50% (depending on the country) of murders of women are committed by their intimate partners [7].

DV is gendered and women are affected the most; about 80% of all reported DV incidents have women as victims [8]. According to the World Health Organization report “Violence against woman prevalence estimates, 2018” [5], 26–28% of all women between the age of 20–44 years do experience or have experienced domestic violence once in their lifetime; 10–16% of all women between age of 20–44 years do experience or have experienced sexual violence from a current or past partner in the past 12 months [2,5].

There are studies which have shown that young age (childhood till the age of 19) is a risk factor for dental trauma [9,10], often due to accidents. Still, dentists should also consider the presence of DV as one possible cause for this kind of trauma.

Dentists seeing patients on a regular basis have the opportunity to detect potential victims of DV, as victims can present with injuries in the region of head, neck and face. Ochs et al. [11] reported that nearly 95% of all victims of physical violence have these kinds of injuries. Another study of Brazilian origin confirmed this: a total of 37.6% of their victim sample had traumas of the head, neck and face. Teeth were affected in 2% of all victims [12]. Although most DV victims do not have dental-related injuries, dentists can nevertheless be considered frontline responders, since it is likely that some patients they see at their dental practice are victims of DV and may depict signs of injuries in the head and facial region.

Based on these findings, it seems important to assess whether dentists are sufficiently trained to detect the signs and indicators of the presence of violence. Furthermore, we wanted to find out whether dentists feel comfortable to communicate with their patients when they suspect the presence of DV and whether they know how to support DV victims.

Therefore, the aim of this review was three-fold: first, to provide an overview of the existing literature on dentists’ knowledge and attitudes towards DV in their daily practice; second, to assess whether and how DV is taught in dental education regarding the aspects of knowledge, beliefs about DV, screening for victims, documenting suspected cases and communication skills; and third, to give recommendations on how to improve dentists’ competence and knowledge in this topic. The recommendations are for dentists, teachers at dental schools, researchers and everyone involved with DV, directly or indirectly.

## 2. Materials and Methods

### 2.1. Search Strategy

This review followed the “Preferred Reporting Items for Systematic Reviews and Meta-Analyses” (PRISMA) guidelines [13]. The main research aims of this review were to investigate dentists´ role in the detection of DV and to determine how well prepared they feel to support victims of DV. The following PICOs (“Patient/Population or Problem”, “Intervention”, “Comparison” and “Outcome”, as defined by McMaster University in Chicago, USA) [14] were defined.

Our criteria of inclusion were studies on dentists’ attitudes and knowledge about DV, studies on programs in dental schools that provide education on DV, studies on how to recognize possible DV victims, studies on how to support victims and studies on the role of DV indicators, such as injuries in the head and face region, in the detection of DV cases [8,12,15,16,17,18,19,20,21,22,23,24,25,26,27,28,29].

Our criteria of exclusion were: (1) articles not written in English [30]; (2) case reports, letters, conference abstracts and systematic reviews [31]; (3) study designs with less than 5 participants; (4) studies on elder abuse and on children (as DV was defined according to WHO as intimate partner violence [5]).

### 2.2. Selection Process and Data Extraction

**Figure 1:**
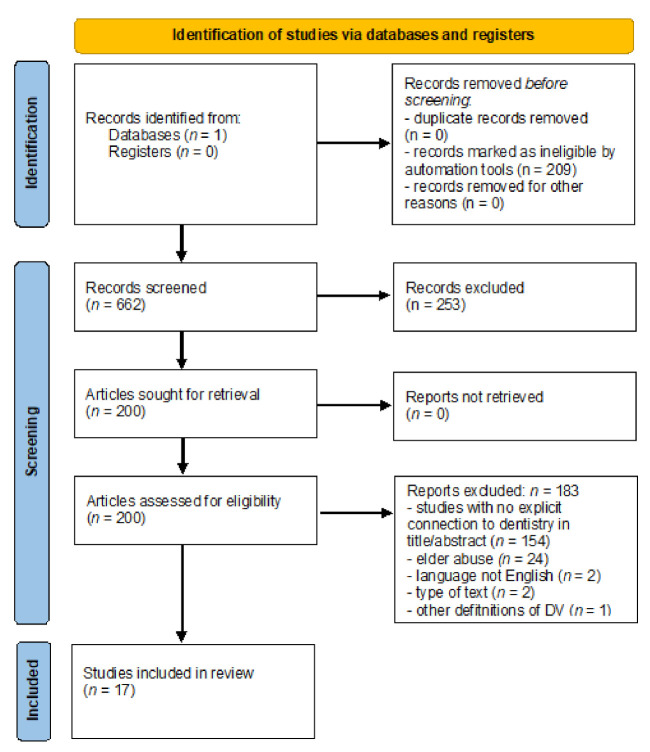
PRISMA 2020 flow diagram for new systematic reviews, which includes searches of databases and registers only [13]. Search process in PubMed based on our chosen keywords. Four steps were performed: identification, screening, eligibility assessment and the final inclusion of papers. Papers were included from the time period of January 2020 to October 2021, starting with 662 papers and finally including 17 according to our defined eligibility criteria.

The online search was performed using the PubMed database. Figure 1 shows all studies identified from January 2020 until October 2021. The following keywords were used for our online search: “domestic violence and dentists”, “abuse and dentists”, “domestic violence and dental schools”, “domestic violence practice dental schools” and “domestic violence face region”.

The studies were selected by one reviewer (JB) and then discussed with a second reviewer (BP) after the analyses of the articles. First, articles were selected by reading their titles and abstracts. After that, the full length article was read if the criteria of inclusion were met. All articles that did not comply with the inclusion criteria were excluded (Figure 1).

All relevant material regarding the study design plus the information needed were extracted and the results were discussed with the second reviewer (BP).

### 2.3. Quality Assessment

Cochrane defined bias as a “systematic error, or deviation from the truth in results“, which can cause an incorrect estimation of effects in a study, and therefore may lead to a wrong result [32].

Due to the different study designs, different tools for risk of bias assessment were used. All tools used were Joanna Briggs Institute (JBI) tools from the University of Adelaide [33,34,35].

## 3. Results

### 3.1. Literature Search

Originally, 662 articles were found. Of those, 209 articles were excluded because the year of publication was before 2000, which was flagged by an automation tool. Furthermore, 253 articles were excluded after screening because they dealt with child abuse. No articles were retrieved. After assessing their eligibility, 183 articles were excluded because they either dealt with elder abuse, used different definitions of DV from the WHO [5], used an excluded study design (Table 1) or were written in a language other than English. In the end, 17 studies were included and used for the review (Figure 1).

### 3.2. Study Characteristics

The studies included were published between 2000 and 2021. After reviewing the 17 available papers, several topics related to DV were identified: injury pattern in the head and facial region in cases of DV, the expectations of DV victims towards dentists, dentists’ level of knowledge about DV and the support of DV victims, dentists’ barriers and fears for not asking about the reasons of injuries and available training for dentists on DV. All the analysed papers are listed in Table 2. Five of the seventeen papers included in this review were cohort studies (29.4%). Boyes [15] investigated the occurrence of maxillofacial injuries associated with DV in his study in 2019. Raja et al. [16] published a study on a two-day training programme for students that dealt with victims of DV in 2015. Gibson- Howell et al. [17] reported on DV training in dental school curricula in 2008. Warburton et al. [18] also developed a training programme on DV for dental hospital staff and analysed the changes in their knowledge and attitudes; their study was published in 2006. Le et al. [19] focused on detecting DV-associated injuries in their 2001 study.

Nine of seventeen studies were cross-sectional studies (52.9%). In 2021, Buchanan et al. [20] described the results of teaching first-year students about DV. AlAlyani et al. [21] published a study about the awareness of and actions toward patients who witnessed DV in 2017. Mythri et al. [22] assessed dentists’ knowledge and barriers when facing DV victims in their 2015 study. Patel et al. [23] performed a study on teaching undergraduate dental students about DV, which was published in 2014. Garbin et al. [12] investigated the different types of DV injuries and published their study in 2012. Drigeard et al. [24] reported on the knowledge and attitudes of French dentists regarding DV in 2012.

In 2009, Nelms et al. [25] described what victims of DV in a dental practice need and what they want as a patient. In 2001, Love et al. [26] reported on the attitudes and behaviours of dentists regarding DV. Van Dam et al. [8] published a study in 2005 about Dutch dentists implementing the DV reporting code of the Dutch Dental Association in their practises and about the experiences dentists had with DV victims.

One of the seventeen studies included in this review was a case series study (5.8%) by Brink et al. [27] that dealt with the collection and description of DV injuries.

McAndrew et al. [28] published a quasi-experimental study about the effectiveness of an online tutorial for dental students in 2014 (5.8% of all included studies).

There was one randomized controlled trial used for the review (5.8% of all included studies). Hsieh et al. [29] also dealt with multimedia training for dentists and how the knowledge and attitudes of the dentists changed.

**Table 2:** Overview of all studies (n = 17) included in this review according to our eligibility criteria (see Figure 1).

Study	Title	Year of Publication	Study Aim
Buchanan et al. [20]	Longitudinal curricular assessment of knowledge and awareness of intimate partner violence among first-year dental students	2021	Outcomes of teaching first year undergraduate dental students topics in DV (see Table 3).
Boyes et al. [15]	Maxillofacial injuries associated with domestic violence: experience at a major trauma centre	2019	Identification of victims of DV and finding out how competent dentists feel about DV.
AlAlyani et al. [21]	Dentists’ awareness and action towards domestic violence patients: a cross-sectional study among dentists in Western Saudi Arabia	2017	Barriers faced by dentists when dealing with DV victims, identification of factors predicting awareness about DV among dentists and factors influencing the action required when facing victims.
van Dam et al. [8]	Recognizing and reporting domestic violence: attitudes, experiences and behavior of Dutch dentists	2015	Assessment of whether general dental practitioners in the Netherlands are aware of the reporting code published by the Dutch Dental Association, if they introduce it into their practice and how often they suspected that a patient was a DV victim and how they dealt with that patient
Mythri et al. [22]	Enhancing the dental professional’s responsiveness towards domestic violence: a cross-sectional study	2015	Knowledge assessment regarding DV among dentists of the region of Karnataka (India), assessing barriers dentists face when seeing victims.
Raja et al. [16]	Teaching dental students to interact with survivors of traumatic events: development of a two-day module	2015	Explanations on how to develop an interactive educational module on DV for dental students and how future education could be structured (see Table 3).
McAndrew et al. [28]	Effectiveness of an online tutorial on intimate partner violence for dental students: a pilot study	2014	Assessment of the effectiveness of an online based tutorial for senior dental students regarding knowledge, attitudes, beliefs and behaviours towards DV (see Table 3).
Patel et al. [23]	Domestic violence education for UK and Ireland undergraduate dental students: a five-year perspective	2014	Outcomes of teaching undergraduate dental students topics in DV in 2007 and 2012 (see Table 3).
Garbin et al. [12]	Occurrence of traumatic dental injury in case of domestic violence	2012	Investigation of prevalence and types of traumatic dental injuries in DV victims.
Drigeard et al. [24]	Educational needs in the field of detection of domestic violence and neglect: the opinion of a population of French dentists	2012	Knowledge assessment of dentists about DV, information on how dentists should respond to victims, defining expectations that could be used for further education.
Brink et al. [27]	When violence strikes the head, neck and face	2009	Systematic collection, analysis and description of injuries by victims of DV, differences in sex and location of injuries.
Nelms et al. [25]	What victims of domestic violence need from the dental profession	2009	Assessment of the impact of race, age, sex on the prevalence of DV; location of injuries; experiences with DV at a dental practice; and the needs of victims when facing members of the dental profession.
Gibson- Howell et al. [17]	Instruction in dental curricula to identify and assist domestic violence victims	2008	Assessment of dental schools regarding the inclusion of DV as a teaching topic (see also Table 3)
Warburton et al. [18]	Changes in the levels of knowledge and attitudes of dental hospital staff about domestic violence following attendance at an awareness raising seminar	2006	Assessment of levels of knowledge and awareness about DV among dental health care workers, changes after a seminar on DV.
Hsieh et al. [29]	Changing dentists’ knowledge, attitudes and behavior regarding domestic violence through an interactive multimedia tutorial	2006	Development of a brief tutorial aiming to educate dentists and assessment of the tutorial (see Table 3).
Le et al. [19]	Maxillofacial injuries associated with domestic violence	2001	Identification of patterns in maxillofacial injuries of DV victims.
Love et al. [26]	Dentists’ attitudes and behaviors regarding domestic violence: the need for an effective response	2001	Investigation of attitudes and behaviours of dentists regarding DV, barriers to support victims, encouraging further research on the topic of DV.

**Table 3:** Overview of all included studies (n = 6) on tutorials for dental students with country of study, study design, number of participants, teaching technique and main outcomes before and after the completion of the tutorials.

Study	Title	Year of Acceptance	Countries of Study	Study Design	Number of Participants	Teaching Technique	Main Outcomes	Differences Before and After Training
Buchanan et al. [20]	Longitudinal curricular assessment of knowledge and awareness of intimate partner violence among first-year dental students	2021	USA	Cross-sectional study	n = 232 ^2^	Brief pre- and post- testing, instructional workshop in class.	Before the workshop, two thirds of the students had received no education on DV at dental school. DV as a healthcare issue and knowledge about procedures when a DV victim is identified improved.	Pre-testing: 51.3% stated that DV is a dental healthcare issue, with 61% of the female and 41% of the male students agreeing with this. - Post-testing: 81% thought of DV as a dental healthcare issue, of which 77% were male and 86% female. - Awareness of resources and information about DV rose from 18.1% to 83%.
Raja et al. [16]	Teaching dental students to interact with survivors of traumatic events: development of a two-day module	2015	USA	Cohort study: clinical study of a whole cohort of dental students	n = 92 ^2^, n = 102 ^2^	First module: lecture and discussion. Second module: lecture, handouts, role plays, videos, practises on documenting findings in patient charts.	Competence about the topic of DV increased, knowledge about the importance of reporting and documenting DV improved.	Pre- and post-testing: communication skills and understanding of DV victims improved. Students were still unsure of when to report a potential victim of DV.
Patel et al. [23]	Domestic violence education for UK and Ireland undergraduate dental students: a five-year perspective	2014	UK, Ireland	Cross-sectional study	in 2007: n = 12 ^1^; in 2012 ^1^: n = 11	Lecture, video lessons, group work.	Reasons for not teaching DV: - Lack of time. - Topic not important enough for teachers.	No difference in attitudes towards the topic of teaching DV to dental students.
McAndrew et al. [28]	Effectiveness of an online tutorial on intimate partner violence for dental students: a pilot study	2014	USA	Quasi-experimental study	n = 25 ^1^	Online tutorial, pre- and post-testing.	Possible to change knowledge, but changing beliefs is difficult.	Post-testing: actual and perceived knowledge and preparation for dealing with DV victims significantly improved; only two opinions about DV improved significantly.
Gibson-Howell et al. [17]	Instruction in dental curricula to identify and assist domestic violence victims	2008	USA	Cohort study: two-part survey study design	in 1996 ^1^: n = 55; in 2007 ^1^: n = 25		Most often taught: the role of dentists, signs in behaviour and injures seen in a possible victim and the reporting and referral protocol. Least discussed: impact of DV in general.	Similar results in 1996 compared to 2007, no significant improvements.
Hsieh et al. [29]	Changing dentists’ knowledge, attitudes and behavior regarding domestic violence through an interactive multimedia tutorial	May 2006	USA	Randomised two group controlled trial	n = 174 ^2^	Interactive multimedia tutorial with pre- and post-testing.	Possible to change knowledge, but changing beliefs is difficult.	Post-testing: significant improvement in knowledge, but no change in beliefs and attitudes about victims.

^1^ Dental schools; ^2^ dental students.

### 3.3. Quality Assessment

Cohort Studies (Table 4):

The risk of bias for all the cohort studies used was rated as moderate: the exposures measured were valid and similarly measured in all groups, the participants were independent of the outcome of interest, the outcomes were measured in a valid way and the statistical analyses used were rated as positive. Only questions regarding the follow-up were answered with ‘no’, because there was no description about dropouts, i.e., how many participants did not complete the survey and their reasons for dropping out of the study.

Cross-sectional studies (Table 5):

The results of the cross-sectional studies were also rated as positive for clearly defined samples, the description of study settings, the validation of measurements, the objectivity of criteria for measurements and mostly for the statistics used. Therefore, these studies were also rated with a moderate risk of bias. Only the strategies for confounding factors were rated as unclear in the studies by Nelms et al. [25], Love et al. [26] and van Dam et al. [8].

Case series study (Table 6):

For the case series study, the conditions and inclusion criteria, valid methods for identification and inclusion of participants, the reporting of outcomes, appropriate statistical analyses and the reporting of clinical demographic information were rated as positive. Demographic information about the participants were rated as unclear. Brink et al.’s [27] case series study was rated as having a moderate risk of bias.

Quasi-experimental studies (Table 7):

McAndrew et al. [28] did not have a control group in their quasi-experimental study; therefore, this question was answered with ′no′. The other questions, such as the description of cause and effect, similarity of comparison, similarity of treatment, pre- and post-test measurements, reliability and comparison of outcomes as well as appropriate statistical analysis were answered with ‘yes’. All in all, this study was given a moderate risk of bias.

Randomized controlled trials (Table 8):

The risk of bias for Hsieh et al.’s [29] randomized trial was rated as high. The groups of participants were similar, the participants were analysed in the groups they were randomized to, the outcomes were measured in a reliable way and an appropriate statistical analysis was used; however, it was unclear how the randomisation was performed, whether the participants were blinded to their group and whether the randomized trial was appropriate to provide information. The questions regarding identical order in training and control tests of groups and follow-up were answered ‘no’.

### 3.4. Injury Pattern in the Head and Facial Region

In a study based on police reports by Garbin et al. [12], it was observed that out of 7750 reported violence cases, 1844 were associated with DV. In 15 cases, teeth injuries were part of the injuries reported. The most affected teeth were the maxillary incisors, followed by the mandibular incisors due to their anatomic localization. Boyes et al. [15] documented that out of 176,759 patients seen in 1.5 years at the King’s College Hospital emergency department in London, only 18 were identified as victims of DV. Nearly all the patients had facial lacerations, and half of them had fractures. The most common fractures involved the dento-maxillar part of the face. Similarly, the study by Brink et al. [27] showed that of all DV-associated cases with injuries in the head and facial region in a hospital in Aarhus, Denmark, 41% were women (total 327) who were hurt by either their current or ex-partner. The nose, orbita and mouth region were the most affected. Moreover, 10 out of these 348 women had a teeth injury [26].

In another study, it was reported that of 112 questionnaire respondents, 76 women had experienced violence and injuries in their head, neck and face. The most common location was the lip, followed by the face and neck. Thirty-five women stated that they had broken teeth, and 11 had lost a tooth due to violence [25]. Comparable results were detected in another study where out of 236 women, 81% had injuries located on the face, with soft tissue injuries being the most common. Fractures were mostly located in the middle face [19].

In summary, although injuries of the teeth and oral cavity are rather rare [12,25], signs of DV could nevertheless be detected by a dentist, as the head and face in general are frequent places where victims are injured [15,19,25,27]. The prominent localisation of injuries in the head and facial region of victims of DV negatively impacts victims psychologically, since the face is considered as part of the character and personality of the person; hitting someone in the face is a demonstration of the unequal distribution of power between the victim (lower) and perpetrator (higher) [27].

### 3.5. Expectations of DV Victims towards Dentists

To gain a better understanding what DV victims would need from the dental sector, Nelms et al. [25] analysed a questionnaire that was completed by 85 DV victims. Of those 85, 12 needed to visit a dental clinic because of oral injuries caused by DV [25]; of the 12 female victims, eight were treated by a male and four by a female dentist. Most of the victims had no preference regarding the sex of their treating dentist. Remarkably, only 13.3% percent of all women (n = 83) with injuries of the head and face who visited a dental clinic for routine dental work unrelated to violence some time later were asked about the reason for their injuries. The majority of women (69.3%) stated that they would have wanted to be asked what had happened. Embarrassment and fear were reasons for not wanting to be asked (30.7%). Only half of the women who were asked about their injuries and who had disclosed the presence of DV received some support in form of the phone numbers of shelters and the police [25].

### 3.6. Level of Knowledge of Dentists about DV and Support of DV Victims

Although most dentists are well aware of DV as a serious public health problem, it is still unclear for them when to intervene [21] and they do not know whether they have enough knowledge to do so. Only one of three dental practitioners believed to have had seen a victim of domestic violence in their practise according to one study [21]. This is in line with other reports. Of 151 responders to a questionnaire, 30.5% stated to have treated DV victims in their practise [21]. The same percentage was observed in an Indian study [22] and was also comparable to a Dutch study [8]. Here, approximately 24% of all general dental practitioners who had participated in the survey assumed they had contact with a suspected victim in the last 12 months [8]. This is in line with another recent study [24] with 228 participants; 36% of all general dental practitioners had at least seen one case of DV and 48.2% stated that they had a suspicion of several cases, with most of the victims being women. Knowing that about one in three women do experience DV in their lifetime [2,5], it is highly unlikely that 60% of all dentists have never treated a DV victim at their dental practice.

When being asked about screening patients for DV, 87% of 321 responders to a questionnaire in another study never screened new patients for DV, and 85% never screened patients at regular check-ups [24]. The screening rate was slightly higher in a study by AlAlyani et al. [21]; however, still only 49.6% of the 151 respondents indicated that they screen new patients and 46.6% that they screen at check-ups for DV [21]. The screening rate was somewhat higher if injuries in head and facial regions were noticeable; still, less than half of all responders regularly screened the victims [24], even though they were aware that DV can cause these kinds of injuries in these areas [21,22]. After DV in patients was disclosed, dentists mostly took notes in the patient′s chart but very few expressed their concerns for the victims’ safety and provided information about shelters and contact points for persons affected by domestic violence [8,21,24].

### 3.7. Barriers and Fears of Dentists for Not Asking about the Reasons of Injuries

The most common reasons for not asking victims about the cause of their injuries included situations where victims were not alone but accompanied by children or partners, the lack of training in identifying victims, and the embarrassment of talking about DV with the fear of offending the patient/victim [8,21,22,24]. Another problem was that the practitioners themselves did not have enough information about agencies that might help the victims [21,22,24,26]. Dentists who never had experience with DV victims previously found it more difficult to help victims, whilst dentists with more experience were more likely to discuss the patient’s case with other healthcare colleagues. Of 228 dentists, 53.3% said that they feared the consequences for their patient in the case of intervening and also nearly half of them were not familiar with the existing laws and obligations regarding reporting abuse [24]. Besides that, a lack of time to discuss the issue of DV during patient visits seems to be an additional barrier [22,26]. Less of a barrier seemed to be that dentists considered DV as being none of their business, as 64% of 536 responders to a questionnaire were aware of the important role of dentists in tackling DV [22]. Over all, most dentists, more female then male (39% vs. 24%) [8], reported that they would like to educate themselves more in the topic of DV and how to detect it in order to help victims [8,18,22].

### 3.8. Available Training on DV for Dentists

More than half of all dentists do not receive any training on how to detect and how to interact with possible victims, neither at dental school nor in further trainings [24,26]. This can be considered a missed opportunity, as studies have indicated that even a short training intervention can help participants of other health care sectors in improving their knowledge and behaviour towards victims [18].

One of the few available studies regarding training on intimate partner violence in dental schools was published by Patel et al. [23] in the UK; the authors compared how many dental schools taught their students about DV in the years 2007 and 2012. In 2007 [23], out of the 12 schools that responded to the questionnaire, only six had DV in their curricula, mostly in the surgery or paediatric dentistry classes. The most common teaching technique was a simple lecture; however, video lessons and working in break-out groups were also used. Among the schools not teaching DV, five stated that they had no interest in including the topic in their curricula.

The reasons for these responses were either teachers’ lack of time or lack of knowledge regarding the importance of the topic. In 2012, only five of the 11 responding dental schools were teaching about DV; one of the schools had a whole module that thematized the topic [23]. Compared to 2007, the methods of teaching did not change much, with lessons up to 4.5 h in length. The reasons given by the four out of six schools who were not willing to include teaching about intimate partner violence to their curricula were the same as in 2007. All in all, it was reported that in the time period studied, not many changes were detected [23].

Another survey by Gibson-Howell et al. [17] was performed in the US in 1996 and 2007. A questionnaire was sent to dental schools to investigate whether and in what topics dental students were taught about DV. Additionally, beliefs towards DV were assessed. The rate of responses regarding the first questionnaire in 1996 was nearly 86% of the 64 schools who received the questionnaire. The most commonly taught DV topic (n = 31) was the role of the dentist, followed by DV signs in behaviour and external appearance, DV reporting and referral protocol and DV prevalence. In 2007 [17], only twenty-five of fifty-five dental schools answered the questionnaire. Nevertheless, the results looked similar to those from 1996 [17]: the role of dentists was again the most commonly taught topic, followed by the characteristic signs of injuries/external appearance and the behaviour of victims and the reporting/referral protocol. Training about options on how to improve the victim’s situation was the least discussed topic of all eleven listed (n = 3). Regarding the statements about beliefs that were listed, the responses given did not differ between 1996 and 2007. Most agreed with the statement that good patient relations combined with good communication skills may be the key to recognizing victims of intimate partner violence. The other statements were also highly agreed on, apart from “DV is an increasing health care issue”, which only obtained an agreement rate of 60% [17].

In a pilot study conducted in New York University, College of dentistry [28], a total of 25 senior dental students participated in an online tutorial consisting of a pre- and post-test with a duration of 1.5 h as part of a control or treatment group. When comparing the results of the pre- and post-tests, it was found that the actual and perceived level of knowledge, as well as feeling well prepared to deal with DV victims, were significantly higher in the post-test. Regarding the opinion questions used in the tests, only two opinions—“self-efficacy” and “constraint”—improved significantly after the tutorial.

Both, McAndrew et al. [28] and Hsieh et al. [29] stated that it seems possible to change knowledge, but changing beliefs about DV is more difficult. This was confirmed by another pre- and post-test study where two different randomized groups of dentists (control and intervention group) were surveyed. The control group (n = 88) first took the pre- and post-test and then had the tutorial about DV; the intervention group (n = 86) took the pre-test first, then had the tutorial and took the post-test afterwards. After the tutorial, all questions about how to help the suspected victims of DV and about the knowledge regarding the topic of intimate partner violence victims in dental practices had improved in the intervention group. The results were not as effective regarding the beliefs and attitudes about victims in dental practices. Compared to the control group, the changes were only significant in four of twelve beliefs.

The University of Illinois, Chicago developed a training about interpersonal trauma victims, including DV victims, in dental practises in two steps [16]. The first module contained information about how victims may behave and the injuries that may be seen immediately after the trauma and after some time has passed. Moreover, it provided information about the best timeframe for dentists to check for violence and how to report suspected cases. This module had a duration of three hours and was re-evaluated afterwards with improvement suggestions from the participants, which were second-year dental students [16]. The revised and further optimised module was completed in two sessions afterwards (3.5 h each) by second-year dental students. Topics that were added included information about communications, collaborations with other institutions and addresses that might be useful to support victims, screening for potential victims, and behavioural and reaction training for when victims talk about their history. There was role-playing in small groups to practice communication skills, with feedback given by the other participants of the group; videos of victims and their stories; a training on how to document cases; and role-play training with an actor who played a DV victim [16]. Before and after the training, the students took part in a test to document how effective the training was regarding communication skills and the understanding of the victim’s needs. Students´ degree of feeling comfortable and competent in dealing with DV increased significantly after the training. Additionally, knowledge about the importance of reporting and documenting cases improved [16]. Nevertheless, the tests showed that the students were still unsure about whether DV was present and when to report it, indicating the length of the training was not sufficient.

A recent study performed in 2021 at the University of Nevada [20] revealed that a short training for first-year dental students was very successful. A total of 232 participants went through an in-class instructional workshop with integrated pre- and post-testing; 62.5% of the 232 students were male. Most attendees were of white ethnicity. Of all the students, 64% stated that they had not received any formal education about intimate partner violence before at dentistry school. Regarding DV as a healthcare issue, students’ opinion changed after the workshop. Previously, only 51.3% thought that DV was a dental healthcare worker’s issue. An interesting point is that more female participants (69%) agreed with the above statement, whilst only 41% of all male participants agreed. The post-test results showed an 81% agreement with the statement that DV is an important topic in dentistry (77% were males and 86% were females). Moreover, knowledge about resources and procedures after identifying a patient as a victim of DV improved from pre-testing considerably, from 18.1% to 83%. All papers about the impact of training are listed in Table 3.

## 4. Discussion

Our literature review revealed that many hurdles exist to adequately identifying and supporting potential DV victims in the dental sector: a lack of knowledge about indicators of DV, a lack of knowledge on how to document detected cases and a lack of knowledge on how to help victims has resulted in uncertainty about the topic of DV among dentists [21,22,24,26]. Dentists often had wrong beliefs about victims of DV [29] and most had never received any formal training regarding DV [21,22,24,26] (Table 2 and Table 3).

There is evidence to suggest that one out of three women suffer from DV [2,5]. Indicators for the presence of physical violence are injuries in the head, neck and face area [15,19,25,27], although not necessarily in the oral cavity as teeth are rarely affected [12,25]. The reasons why head, neck and face injuries are more common in physical violence are the easy access as well as the psychological factor that emphasises the superiority of the perpetrator [27]. Despite the high prevalence of DV, the numbers of victims detected in dental practices are comparatively low [21,22,24], which can be explained in part by the low frequency of regular screenings for DV and the lack of training on DV [8,21,22,24].

The majority of dentists are aware that they will most likely see DV victims in their dental practise; however, they do not feel sufficiently competent in helping victims due to a lack of knowledge [21]. Moreover, they do not feel comfortable enough asking their patients about DV. The reasons provided for not asking were that the victims were either accompanied by family and/or that the dentists were afraid of offending the victim; in addition, a lack of information about what to do after the victim disclosed that they were suffering from violence presented another obstacle to asking [8,21,22,24]. This, too, can be attributed to a lack of training, as many dentists are not informed about their reporting duty [24]. Even in cases where DV was suspected, a record in the patients’ file was made, but victims did not receive any information about shelters and/or contact points for DV victims [8,21,24,25]. DV victims clearly want to have more support [25].

The importance of DV as a public health problem has not been questioned [16,17,29] and the statement that dentists have an important role in detecting victims has reached strong agreement [29]. The belief that victims do not appreciate the support they are given and that victims will stay with their perpetrator even if they are supported by others did not change even after a training programme [29]. However, even if beliefs regarding DV did not change significantly [29], most who participated in the trainings felt more competent in understanding the topic and talking to the victim after they had received some form of training [16].

In all studies on DV training, the impact was found to be positive, as knowledge and confidence in dealing with cases where DV is suspected increased significantly among the participants of trainings [16,17,20,28,29]. Therefore, it is problematic that very few dental schools have courses on DV in their curricula [23]. The reasons given for this were a lack of time for this topic in the curriculum and a lack of knowledge regarding the topic of DV [23], which shows that DV needs more attention in the dental health sector.

In those dental schools where DV was taught, most teaching formats included some form of an online teaching session (Table 3). Only Raja et al. [16] reported that they used role-playing with actors and videos of real victims to enhance the impact of the tutorial, which was also very well received by the attending dental students. Role-playing may encourage more participants to engage with the topic and improve their knowledge.

Regarding the gap in knowledge and the lack of mandatory DV training, there is no major difference between dentists and medical doctors. Studies have shown that there is also a lack of training for medical doctors and a lack of knowledge on how to deal with victims [36]. In addition, many physicians do not know how to interact with special victim groups or have often overlooked males [37] and/or children [38] as victims and do not know how to support them well. It has been shown that medical doctors, similar to dentists, are often too afraid to ask patients about DV [39]. A lack of time and not feeling competent to identify and support victims seems to be the major hurdles for medical doctors in taking further steps after DV has been disclosed [39]. DV is not a mandatory topic among Europe′s medical profession groups [39], which needs to be changed.

Looking at the studies available (Table 3), it is noticeable that no long-term effects of training interventions have been investigated [16,17,20,23,28,29]. It is generally known that teaching must be repeated several times to make it sustainable [40]. In line with this, the topic of DV should not only be taught in one single tutorial, which only takes few hours, but should be included in several lectures at university dental classes. This would also help to reduce the barriers to addressing victims and strengthen competence to document DV cases properly [16,21,22,24,26].

Although most studies on teaching dental students about DV have been performed in the USA, with only a few in Europe and one in India, the main results are strikingly similar. Regarding DV as a worldwide health system problem, it would be interesting to investigate the situation regarding DV education in other countries in order to share best-practice examples and to learn from each other. This would also raise the number of victims detected globally.

As one limitation, it is important to note that the number of participants enrolled in the studies discussed here was rather low. This may be a sign that the topic is not considered important enough to students and teachers alike [23]. In addition, more general information about participants, e.g., age, gender and origin/ethnicity, should be investigated to assess whether opinions are dependent upon the educational status, social classes, age groups, etc., of the participants.

A further limitations is the study designs: as the designs differed in the available studies, it was difficult to compare the studies in a statistical way. Furthermore, the quality assessment regarding the risk of bias was more complex, as many different risk-of-bias tools were needed to compare the quality of all the included studies. More randomized studies about documenting the educational process would facilitate a comparison and ensure a higher quality of the results.

Randomization needs to be well planned and documented to provide a good quality study with a low risk of bias. Moreover, more information about drop-out rates are needed, as the follow-up in the reviewed studies was not always completed [15,16,17,18,19]. As mentioned above, no long-term effects of trainings could be assessed, as the follow-up was not long enough to evaluate whether the training interventions had a sustainable impact on the behaviour and knowledge of the dentists/students participating in the study.

## 5. Recommendations

Based on the results of our review, we propose the following recommendations:

1. Domestic violence as a part of the formal education of dentists is an important factor in the prevention of DV and should be mandatory teaching at dentistry schools and in further training for dentists on a regular basis using innovative training concepts, such as role-playing. The impact of these interventions should be investigated in further studies.
2. An important focus should be put on communication with victims of DV, as dentists do not feel competent at all in this area. The following aspects should be covered and included in trainings:
  Make sure that the patient/victims are alone and can speak freely. Often victims are accompanied by either children or even partners [41,42]. Ask general questions about the state of a suspected victim′s relationship to start the conversation, e.g.,: “How are you getting along with your partner?“ ”How are things at home?” [42] If you feel the suspected victim wants to talk about their experience, direct questions might be asked, such as: “Have you ever been verbally/physically/emotionally/sexually abused by your partner?” [41,42]
3. Established guides, e.g., the R.A.D.A.R. acronym [43], which was developed by the Massachusetts Medical Society [43] in 1992, should be used (Table 9).
4. Materials from online training platforms, for example, the IMPRODOVA training platform, on DV with case studies, statistics, presentations and quizzes [44] for teaching should be incorporated into trainings. The latter is a living document and will be updated on a regular basis.
5. As almost all articles on DV training are from the US or UK, studies on trainings should be conducted in other countries to improve the local detection and local support of DV victims.

## 6. Conclusions

To conclude, although the number of DV victims with dental injuries in dental practices is not high, knowing that one in three women suffer from DV clearly indicates that many victims go undetected. In this sense, the low rate of screening questions for new dental patients, especially when signs of facial injury are present, is of concern and clearly supports our call for formal training on DV.

## Figures and Tables

**Table 1:** PICO model as defined by the McMaster University of Chicago [14].

Focused Question	
PICO model	How good is the knowledge (O) of the dentists (P) in recognizing and intervening with patients who are victims of domestic violence (I)?
**Search Query**	
	#1 (domestic violence) OR (abuse)
	#2 (domestic violence) AND (face region)
	#3 (dentists) or (dental school)
Study design	No case reports, letters, conference abstracts and systematic reviews

**Table 4:** Risk of bias assessment for cohort studies according to the JBI tool (sorted in order of year of publication) [33].

Study	Q1	Q2	Q3	Q4	Q5	Q6	Q7	Q8	Q9	Q10	Q11	% Yes	Risk of Bias
Boyes [15]	Yes	Yes	Yes	No	Yes	Yes	Yes	Not applicable	Not applicable	Not applicable	Not applicable	54.5	Moderate
Raja et al. [16]	Yes	Yes	Yes	No	Yes	Yes	Yes	Not applicable	Not applicable	Not applicable	Yes	63.6	Moderate
Gibson-Howell et al. [17]	Yes	Yes	Yes	No	Yes	Yes	Yes	Yes	No	No	Yes	72.7	Moderate
Warburton et al. [18]	Yes	Yes	Yes	No	Yes	Yes	Yes	Yes	No	No	Yes	72.7	Moderate
Le et al. [19]	Yes	Yes	Yes	No	No	Yes	Yes	Not applicable	Not applicable	Not applicable	Yes	54.5	Moderate

**Table 5:** Risk of bias assessment for cross-sectional studies according to the JBI tool (sorted in order of year of publication) [33].

Study	Q1	Q2	Q3	Q4	Q5	Q6	Q7	Q8	% Yes	Risk of Bias
Buchanan et al. [20]	Yes	Yes	Yes	Yes	No	Yes	Yes	Yes	87.7	Moderate
AlAlyani et al. [21]	Yes	Yes	Yes	Yes	No	Yes	Yes	Yes	87.7	Moderate
van Dam et al. [8]	Yes	Yes	Yes	Yes	No	Unclear	Yes	Yes	75	Moderate
Mythri et al. [22]	Yes	Yes	Yes	Yes	No	Yes	Yes	Unclear	75	Moderate
Patel et al. [23]	Yes	Yes	Yes	Yes	No	Yes	Yes	Not applicable	75	Moderate
Garbin et al. [12]	Yes	Yes	Yes	Yes	No	Yes	Yes	Yes	87.7	Moderate
Drigeard et al. [24]	Yes	Yes	Yes	Yes	No	Yes	Yes	Yes	75	Moderate
Nelms et al. [25]	Yes	Yes	Yes	Yes	No	Unclear	Yes	Yes	75	Moderate
Love et al. [26]	Unclear	Yes	Yes	Yes	No	Unclear	Yes	Yes	62.5	Moderate

**Table 6:** Risk of bias assessment for case series studies according to the JBI tool [34].

Study	Q1	Q2	Q3	Q4	Q5	Q6	Q7	Q8	Q9	Q10	% Yes	Risk of Bias
Brink et al. [27]	Yes	Yes	Yes	Yes	Yes	Unclear	Yes	Yes	Yes	Yes	90	Moderate

**Table 7:** Risk of bias assessment for quasi-experimental studies according to the JBI tool [35].

Study	Q1	Q2	Q3	Q4	Q5	Q6	Q7	Q8	Q9	% Yes	Risk of Bias
McAndrew et al. [28]	Yes	Yes	Yes	No	Yes	Yes	Yes	Yes	Yes	88.8	Moderate

**Table 8:** Risk of bias assessment for randomized controlled trials according to the JBI tool [35].

Study	Q1	Q2	Q3	Q4	Q5	Q6	Q7	Q8	Q9	Q10	Q11	Q12	Q13	% Yes	Risk of Bias
Hsieh et al. [29]	Unclear	Unclear	Yes	Unclear	Yes	No	No	No	Yes	Yes	Yes	Yes	Unclear	46	High

**Table 9:** Explanation of the R.A.D.A.R acronym with examples [43].

R	Remember to screen routinely	Interview patients regularly if any case of violence occurred
A	Ask	Direct and general questions as mentioned above might be used
D	Document	Taking pictures, documenting details and the victim’s reporting in the patient′s chart
A	Asses the patient′s safety	Asking about weapons, children involved and the patient′s feelings about going home [43]
R	Review available options	If there is a direct risk, the victim should get in touch with a shelter, should receive the phone numbers of support groups and should be offered follow-up appointments to check up on the victims′ well-being.

## Data Availability

The data included in this study are available upon request.
